# Postoperative anticoagulation management using subcutaneous unfractionated heparin for a patient with nonbacterial thrombotic endocarditis: a case report

**DOI:** 10.1093/jscr/rjae215

**Published:** 2024-04-08

**Authors:** Hiroki Mine, Kosuke Saku, Kazuyoshi Takagi, Shoichiro Nohara, Shinichi Hiromatsu, Yoshihiro Fukumoto, Eiki Tayama

**Affiliations:** Division of Cardiovascular Surgery, Department of Surgery, Kurume University School of Medicine, 67 Asahimachi, Kurume 830-0011, Japan; Division of Cardiovascular Surgery, Department of Surgery, Kurume University School of Medicine, 67 Asahimachi, Kurume 830-0011, Japan; Division of Cardiovascular Surgery, Department of Surgery, Kurume University School of Medicine, 67 Asahimachi, Kurume 830-0011, Japan; Division of Cardiovascular Medicine, Department of Internal Medicine, Kurume University School of Medicine, 67 Asahimachi, Kurume 830-0011, Japan; Division of Vascular Surgery, Kurume University Medical Center, 155-1 Kokubumachi, Kurume 839-0863, Japan; Division of Cardiovascular Medicine, Department of Internal Medicine, Kurume University School of Medicine, 67 Asahimachi, Kurume 830-0011, Japan; Division of Cardiovascular Surgery, Department of Surgery, Kurume University School of Medicine, 67 Asahimachi, Kurume 830-0011, Japan

**Keywords:** nonbacterial thrombotic endocarditis, disseminated intravascular coagulation, bioprosthetic valve, anticoagulation, warfarin, subcutaneous unfractionated heparin

## Abstract

Nonbacterial thrombotic endocarditis (NBTE) presents nonbacterial vegetation on cardiac valves. NBTE requires appropriate anticoagulant therapy to prevent recurrence after surgery. However, there has not yet been established evidence for anticoagulant therapy in NBTE, and low molecular weight heparin is not approved in Japan. We present a case of NBTE that was successfully managed with anticoagulant therapy using subcutaneous unfractionated heparin. A 59-year-old woman was diagnosed with NBTE on the mitral and tricuspid valve associated with breast cancer, underwent valve replacement. Warfarin and continuous intravenous unfractionated heparin were started. However, disseminated intravascular coagulation occurred after heparin was discontinued. Continuous intravenous unfractionated heparin injection was resumed immediately, and subcutaneous unfractionated heparin was administered before discharge. Postoperative echocardiography revealed no vegetation on the prosthetic valves thereafter. Subcutaneous unfractionated heparin therapy is useful to prevent the recurrence of NBTE as the anticoagulation in outpatients.

## Introduction

Nonbacterial thrombotic endocarditis (NBTE) is a disease characterized by the presence of nonbacterial vegetation on cardiac valves [1]. NBTE is associated with hypercoagulable conditions such as malignancy and autoimmune diseases [2].

There is a lack of consensus on postoperative anticoagulation, because warfarin should not be used in patients with NBTE [3]. Furthermore, low molecular weight heparin was not approved in Japan. There are no clinical trials comparing the effectiveness of unfractionated heparin and direct oral anti-coagulants (DOACs) in NBTE. We report a case of NBTE that was successfully managed with bioprosthetic valve replacement and anticoagulant therapy using subcutaneous (SC) unfractionated heparin.

## Case report

A 59-year-old woman with stage IV breast cancer was referred to our hospital because of shortness of breath. She had a history of deep venous thrombosis and had been taking DOACs. However, the patient experienced a stroke nine months prior despite taking DOACs.

**Figure 1:**
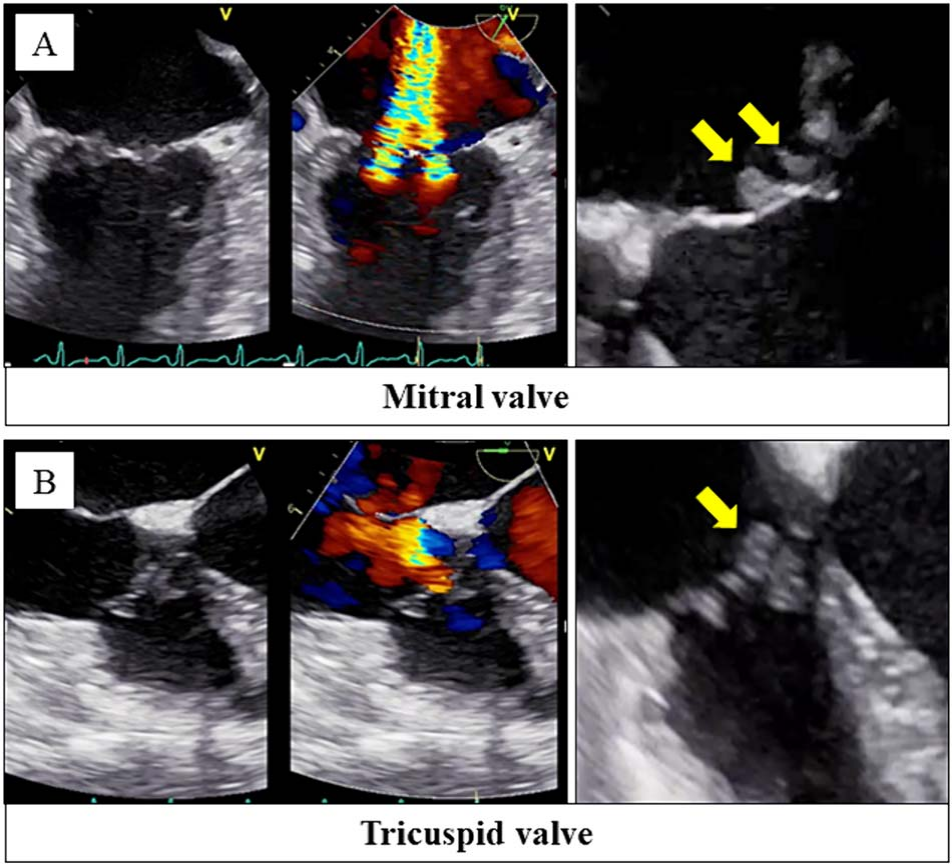
Transesophageal echocardiography revealed severe mitral regurgitation and tricuspid regurgitation with mobile vegetations on both valves (A, B).

Her body temperature was 37.0°C. The laboratory test showed a white blood cell count of 7500 /μl, C-reactive protein of 1.07 mg/dl, platelet of 2.0 × 10^4^ /μl, prothrombin time - international normalized ratio (PT-INR) of 3.47, fibrin degradation products of 66.9 μg/ml, and D-dimer of 21.4 μg/ml. Transthoracic echocardiography and transesophageal echocardiography revealed severe mitral and tricuspid regurgitation with mobile vegetations (Fig. 1A and B).

Although she was afebrile and had no evidence of infection, she was diagnosed with disseminated intravascular coagulation (DIC) due to infectious endocarditis based on echocardiographic findings. Antibiotic therapy and continuous intravenous (CIV) unfractionated heparin were administered for a month. However, echocardiographic findings revealed that the grade of mitral and tricuspid valve regurgitation due to vegetation did not change. In addition, no bacteria were detected in the three sets of blood cultures. We clinically diagnosed the patient with NBTE and coagulation abnormalities associated with the advanced breast cancer. We decided to perform surgery for NBTE.

**Figure 2:**
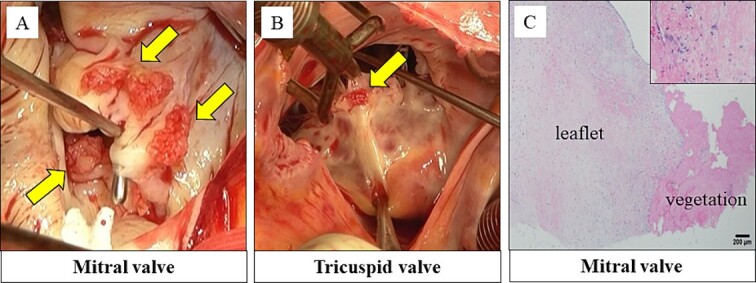
Vegetations were observed on the anterior and posterior leaflet of the mitral valve (A). Vegetations were also observed on the anterior and septal leaflet of the tricuspid valve (B). Histopathological findings: the vegetations on the mitral and tricuspid valves consisted of fibrin and platelets. No bacterial colonies were detected (C).

The vegetation was observed at the coaptation zone on the anterior and posterior leaflets (Fig. 2A). We performed mitral valve replacement with a bioprosthetic valve. Vegetations were also observed on the anterior and septal leaflets of the tricuspid valve (Fig. 2B). The tricuspid valve was replaced with a bioprosthetic valve.

**Figure 3:**
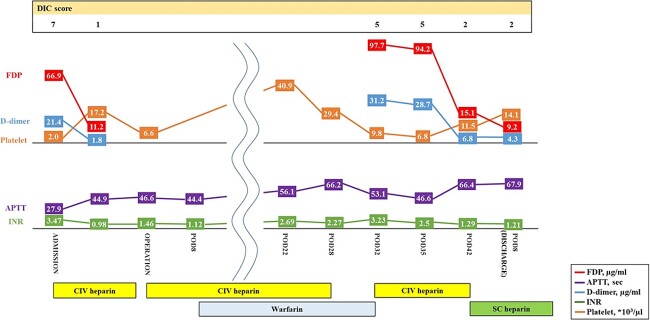
Preoperative and postoperative anticoagulation therapy for nonbacterial thrombotic endocarditis. DIC, disseminated intravascular coagulation; APTT, activated partial thromboplastin time; INR, prothrombin time-international normalized ratio; CIV unfractionated heparin, continuous intravenous unfractionated heparin; SC unfractionated heparin, subcutaneous unfractionated heparin.

**Figure 4:**
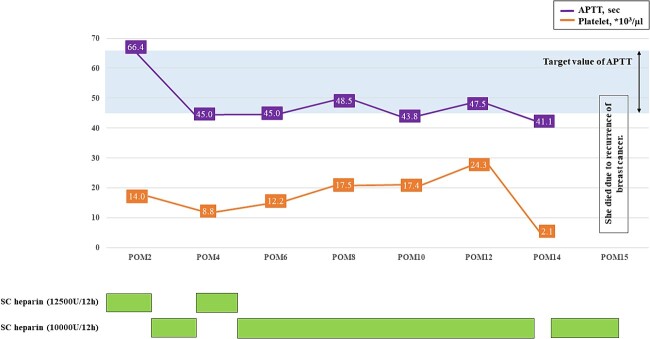
The course of APTT values after discharge. APTT, activated partial thromboplastin time; POM, postoperative month; SC unfractionated heparin, subcutaneous unfractionated heparin.

Histopathological examination revealed that the vegetations consisted of fibrin and no bacterial colonies (Fig. 2C). The patient was diagnosed with NBTE. CIV unfractionated heparin was started on the postoperative Day (POD) 1 and warfarin was started on POD 8, aiming for an activated partial thromboplastin time (APTT) of 45–65 s and PT-INR of 2.5–3.0. However, DIC recurred after heparin was discontinued. We resumed CIV unfractionated heparin injection (Fig. 3). We introduced SC unfractionated heparin and continued it at 10 000–12 500 units per 12 h for self-management before discharge, aiming for an APTT of 45–65 s (Figs 3 and 4). As treatments for breast cancer, hormone therapy and additional molecular targeted therapy were introduced.

Despite the treatment for breast cancer, tumor markers began to increase again, and the patient died of respiratory failure due to hemorrhagic malignant pleural effusion 15 months after surgery without any recurrence of NBTE.

## Discussion

Postoperative anticoagulation and treatment of the underlying disease are important factors for preventing recurrent NBTE. The previous report did not recommend warfarin [3] because the mechanism of hypercoagulation with cancer is multifactorial and the non-vitamin K-dependent pathway (e.g activated tissue factor, V/VIII/X factors, mucin, cytokines, and plasminogen activator inhibitor-1) may induce thrombotic coagulopathy [4]. The most effective anticoagulant therapy for NBTE is considered to be CIV unfractionated heparin or SC low molecular weight heparin. They inhibit thrombin which stimulates the final production of the coagulation cascade, and activates factor Xa which stimulates the production of thrombin [3]. In patients with cancer-associated venous thromboembolism, low molecular weight heparin was more effective than vitamin K antagonist in reducing the risk of recurrent thromboembolism [5]. However, low molecular weight heparin is not approved as a treatment for thromboembolism in Japan. Although DOACs have been proven to be noninferior to low molecular weight heparin in preventing the recurrence of cancer-associated venous thromboembolism [6], in this case, DOACs were considered to carry a risk of recurrent NBTE because stroke and NBTE occurred despite receiving DOACs. Then, CIV unfractionated heparin was initiated on POD1 and continued during the early postoperative period to reinforce the anticoagulation therapy. We selected warfarin as a maintenance therapy because it is an established anticoagulation therapy for outpatients. However, DIC occurred after CIV unfractionated heparin was discontinued despite well-controlled anticoagulation therapy using warfarin. This result suggests that warfarin is not suitable for NBTE. We then introduced SC unfractionated heparin as a maintenance therapy. Although SC unfractionated heparin via self-injection has some problems, such as non-adherence to prescribed medications, subcutaneous hematoma, and fat necrosis, these complications did not occur during the 15 months postoperatively with appropriate self-injection training before discharge.

We concluded that SC unfractionated heparin via self-injection is useful as a postoperative anticoagulant therapy in the outpatient management of patients with NBTE. Warfarin may not be suitable for NBTE.
